# Unveiling the Hidden Bat Diversity of a Neotropical Montane Forest

**DOI:** 10.1371/journal.pone.0162712

**Published:** 2016-10-05

**Authors:** Gloriana Chaverri, Inazio Garin, Antton Alberdi, Lide Jimenez, Cristian Castillo-Salazar, Joxerra Aihartza

**Affiliations:** 1 Universidad de Costa Rica, Golfito, Costa Rica; 2 Dpt. Zoology and Animal Cell Biology, University of The Basque Country UPV/EHU, Leioa, The Basque Country; 3 Natural History Museum of Denmark, University of Copenhagen, Copenhagen K, Denmark; 4 Universidad Nacional de Costa Rica, Heredia, Costa Rica; Università degli Studi di Napoli Federico II, ITALY

## Abstract

Mountain environments, characterized by high levels of endemism, are at risk of experiencing significant biodiversity loss due to current trends in global warming. While many acknowledge their importance and vulnerability, these ecosystems still remain poorly studied, particularly for taxa that are difficult to sample such as bats. Aiming to estimate the amount of cryptic diversity among bats of a Neotropical montane cloud forest in Talamanca Range—south-east Central America—, we performed a 15-night sampling campaign, which resulted in 90 captured bats belonging to 8 species. We sequenced their mitochondrial cytochrome c oxidase subunit I (COI) and screened their inter- and intraspecific genetic variation. Phylogenetic relations with conspecifics and closely related species from other geographic regions were established using Maximum Likelihood and Bayesian inference methods, as well as median-joining haplotype networks. Mitochondrial lineages highly divergent from hitherto characterized populations (> 9% COI dissimilarity) were found in *Myotis oxyotus* and *Hylonycteris underwoodi*. *Sturnira burtonlimi* and *M*. *keaysi* also showed distinct mitochondrial structure with sibling species and/or populations. These results suggest that mountains in the region hold a high degree of endemicity potential that has previously been ignored in bats. They also warn of the high extinction risk montane bats may be facing due to climatic change, particularly in isolated mountain systems like Talamanca Range.

## Introduction

Mountain environments represent one of the most intriguing ecosystems on Earth. The drastic variation of environmental conditions across the elevational gradient [1] promotes major differences between the communities of high mountain areas and nearby lowland sites [2–4]. The specialized traits required to thrive in such environments and/or competition with lowland organisms promote isolation and, eventually, speciation [5–7]. Consequently, most of the endemics to these ecosystems tend to be confined to very small areas within a single mountain or a few mountain ranges, generating assemblages with disjoint distributions [8,9]. In light of the above, the protection of these sites is critical when global warming threatens to shift the typical environmental conditions of highland habitats upward, reducing even more the effective area of many mountain specialists [10,11].

Despite their importance as biodiversity hotspots and centers of endemism [12–17], biogeographic surveys in montane ecosystems are still rare, particularly in regions where these environments are difficult to access. The problem is further exacerbated when considering taxonomic groups that require challenging sampling methodologies. For example, while patterns of diversity and distribution for mountain birds are well known [2,18,19], the knowledge on small mammals such as bats is very limited [20–26]. The scarcity of information about mountain bat communities is partially explained by the demanding fieldwork together with the elusive behavior of these animals, which generally result in a lower sampling efficiency [18,22,27,28] and a diminished interest of researchers on these communities. There is also a good number of studies that have reported an inverse relation between elevation and species diversity [2,4,18,22,29], which may further discourage research in highland sites.

Although understanding evolutionary histories of the organisms that occupy montane habitats is critical for addressing extinction risk under different climate change scenarios, very few studies have examined the evolutionary history of the unique species and species assemblages that inhabit tropical mountain environments (but see [9]). Species with high mobility, such as birds and bats [12,18,30], tend to have wider distributions than those with low mobility [31–33]. Accordingly, several authors stated that, in general, bats of mountain areas should show low levels of endemicity (e.g., [24,25,34]). Nevertheless, recent biogeographic studies demonstrated that bats from tropical montane regions exhibit narrower elevational extents than those of temperate regions, suggesting greater opportunities for isolation and allopatric speciation [35,36]. Besides, the implementation of molecular tools is surfacing the species level identity of a number of populations in mountain ranges that were previously believed to belong to a single taxonomic unit [37–39], even in highly mobile taxa such as birds [6,40]. Molecular studies unveiling cryptic diversity in bats has also become a constant in the last decade [41–51], also in mountain areas [28,42,52]. Available distribution data in Central American countries [53,54], which have been based primarily on morphological identifications, suggest that bats of montane forests have a wide regional distribution. Nonetheless, genetic studies that confirm species identities may provide a clearer picture of species distribution and a deeper understanding of endemism.

The main goal of our study is to identify the taxonomic diversity of bats of the montane cloud forest at Valle del Silencio (2500 masl), and establish the phylogenetic links with populations in other tropical mountain areas in Central and South America. The study area is located in the Talamanca Mountain Range (Costa Rica). It constitutes the main stepping stone between the Sierra Madre range in northern Central America and the Andes in South America, and is a natural barrier between the Caribbean and Pacific coasts of Costa Rica and Panamá, which turns it into an important center of endemism for many plants and animals, as well as harboring several endangered species [12,37,55–58]. Specifically, we use molecular tools to test the hypothesis that at least some of the bats captured at Valle del Silencio are genetically distinct from conspecific populations or sibling species in other regional mountain ranges or nearby lowlands, and thus worthy of more intense taxonomic study. Our study complements other recent efforts that use genetic tools to assess the accuracy of species identification in Neotropical bat communities [43,44,52,59–61], providing an additional step towards understanding cryptic diversity in this highly diverse taxon and region.

## Materials and Methods

### Study Site

La Amistad International Park is one of the largest, yet the least studied, protected areas in Costa Rica and Panamá. La Amistad comprises 400,929 ha distributed between Costa Rica (49.5%) and Panamá (50.5%). This park was granted a World Heritage Site status by the UNESCO, and protects unique and highly diverse ecosystems that have resulted from a combination of a highly variable elevation (range 80–3,549 masl), diversity of soils, differing weather patterns between the Caribbean and Pacific slopes, and unique topographic elements [62]. At least 11 different vegetation types are known from the park, including tropical moist forests at ca. <500 masl all the way up to subalpine rain paramo at >3300 masl [62].

**Fig 1:**
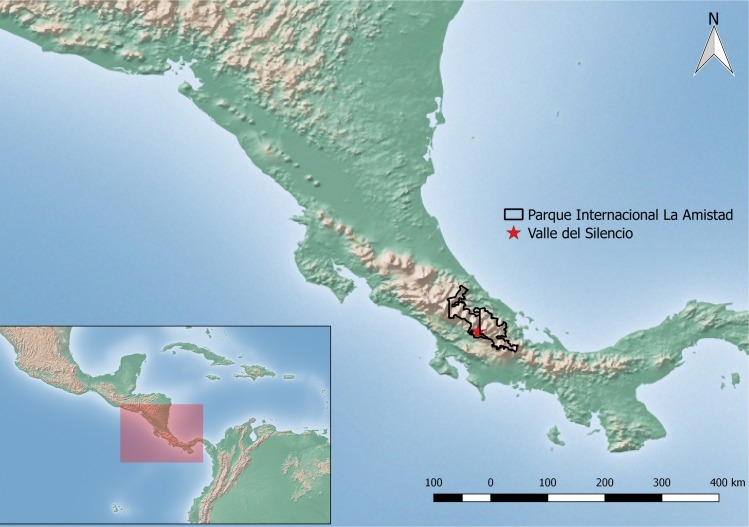
Map showing the location of Valle del Silencio in Parque Internacional La Amistad. Inset shows the enlarged area (red shade) within its geographic context. Data used to generate this map were obtained from Natural Earth (public domain, http://www.naturalearthdata.com/).

The sector known as Valle del Silencio (9°06′42″ N, 82°57′42″ W) is located in the Caribbean slope of the Park yet close to the mountain ridge, at 2,500 masl (Fig 1), and is categorized as a montane rainforest [63]. Temperatures at this site range between 5 and 15°C, relative humidity between 60–100%, and mean annual rainfall is estimated at 4,000–6,000 mm. The only way to access to Valle del Silencio is on foot, and the main hiking trail leading to the area (15 km) starts in the Altamira Ranger Station (1,370 masl), located on the Pacific slope. The canopy vegetation in this valley is dominated by oaks (*Quercus* spp.), whereas the understory is dominated by bamboo (*Chusquea* spp.). Other sites within the valley are dominated by swampy bogs, particularly in the area known as El Jardín (The Garden), where moss (*Sphagnum* spp.) grows abundantly around the fern *Blechnum buchtienii*.

### Bat Sampling and Processing

We placed ground-level mist nets along the main trail leading towards base camp at Valle del Silencio and El Jardin and the surrounding areas. We used a combination of polyester (netting denier/ply = 70/2) and ultra-thin monofilament (netting denier 0.08 mm) nets of 28 mm mesh size (Ecotone, Poland). Monofilament nets were monitored constantly as bats could easily break the threads and escape quickly. Nets varied in size from 3 to 12 m long and 3 m high, and the distance covered by the nets ranged between 47 and 109 m (mean = 73.57, SD = 20.75). Each night, nets were opened from 5:30 pm to 11:00 pm.

Captured bats were released from the nets and stored immediately under the researchers’ clothing for warmth. From each individual we collected standard data, such as forearm length, sex, and reproductive condition, and we got small (3 mm diameter) tissue samples [64] from the wing membrane of 77 live animals using biopsy punches. Species were identified in situ based on general morphology and biometric measurements [61,65], and identifications were subsequently verified using mitochondrial cytochrome c oxidase subunit 1 gene (COI) as a tool for species identification and discovery through the comparison of inter- and intraspecific sequence divergences [44,66]. Sampling within La Amistad National Park was approved by the Costa Rican government (Ministerio de Ambiente y Energía permit numbers R-033-2013-OT-CONAGEBIO and R-057-2015-OT-CONAGEBIO). Bat capture and handling protocols were approved by the Institutional Animal Care and Use Committee of the University of Costa Rica (CICUA-04-14).

With field data of bat species abundance, we estimated species richness [67] and sample coverage (a proxy of inventory completeness [68]), with a combined method of rarefaction and extrapolation using the online version of the software iNEXT [69]. We used 1000 bootstrap runs for constructing 95% confidence intervals up to a sample of 180 individuals, as extrapolation is not reliable beyond double the original sample size [70].

### Molecular Analysis

Fresh tissue samples were stored dried with silica-gel beads until extraction [71]. DNA extraction was carried out with the NucleoSpin Tissue kit (Macherey-Nagel, Düren, Germany; http://www.mn-net.com/) following the manufacturer`s instructions. A 657 bps fragment of the mitochondrial DNA cytochrome c oxidase subunit I (COI) barcode region [66] was PCR-amplified from each DNA extract using two sets of primers and amplification protocols: primers UTyr and C1L705 following [72] and the Mammal Cocktail C_VF1LFt1 + C_VR1LRt1 following [73]. Amplification products were purified with the MultiScreen HTS PCR 96 kit (Millipore, Merck KGaA, Darmstadt, Germany). DNA fragments were bi-directionally sequenced in an automatic ABI PRISM 3130xl Genetic Analyzer sequencer using ABI PRISM™ BIGDYE v3.1 Terminator Sequencing Kit (Applied Biosystems, Foster City, CA, USA).

Forward and reverse sequences were aligned and edited before generating consensus sequences using Geneious v.8.0.5 [74]. DNA sequences obtained for this study were deposited in GenBank (accession numbers KX814389-KX814421; S1 Table) and the Barcode of Life Data Systems (BOLD v3) [75].

Obtained sequences were first compared against the NCBI nr/nt reference database (http://www.ncbi.nlm.nih.gov/) using BLAST (http://blast.ncbi.nlm.nih.gov/Blast.cgi) [76,77]. As a rule, for further comparison analysis we only considered the sequences with a high quality base percentage HQ% above 90. Sequences above that threshold were used to study the phylogenetic relationship of our samples with public sequences belonging to different conspecific populations and other species, obtained from BOLD and GenBank. The public sequences used for comparison are listed in S2 Table. We carried phylogenetic analyses for *Myotis* species and phyllostomids using Maximum Likelihood (ML) and Bayesian inference, as well as median-joining haplotype networks. We used a sequence of *Eptesicus furinalis* as outgroup for the *Myotis* tree, and a sequence of *Artibeus jamaicensis* for the phyllostomid tree. jModelTest v2.1.6 [78] was used to select the best-fitted nucleotide substitution models based on the Bayesian Information Criterion (BIC) values. ML analyses were performed with PhyML 3.0 [79] embedded in Geneious v.8.0.5 [74] with the plugging running PhyML version 3.0 [79], with bootstrap proportions computed after 1000 replicates. Bayesian phylogenetic trees were constructed in MrBayes v3.2.5 [80]. We made two simultaneous runs, with four chains sampled every 500 generations. The program was set to run 10^7^ generations, but to automatically stop when the average standard deviation of split frequencies fell below 0.01. Each Bayesian run was repeated, and convergence of the MCMC chains and sample size was checked using Tracer 1.6.0 to ensure that the effective sample size (ESS) was above 200 and potential scale reduction factor (PSRF) between 1.00–1.02 for all parameters. We discarded the first 25% of generated trees as burn-in. Trees and posterior probabilities were visualized and edited with Figtree v1.3.1 (http://tree.bio.ed.ac.uk/software/figtree/). Median-joining haplotype networks were generated in Network 4.610 (Fluxus Technology, Clare, UK) using the Greedy FHP distance calculation method.

## Results

During 15 nights we conducted a total of 776 mist-net-hours, where a net-hour = 6 m net * 1 hour; 637 net-hours corresponded to monofilament nets, and 139 net-hours to polyester nets. We captured a total of 90 bats, for a capture rate of 0.11 bats per net-hour. In monofilament nets a total of 84 bats were captured, which corresponds to a capture rate of 0.13 bats per net-hour, whereas in polyester nets we captured 6 bats, which corresponds to a capture rate of 0.04 bats per net-hour.

**Fig 2:**
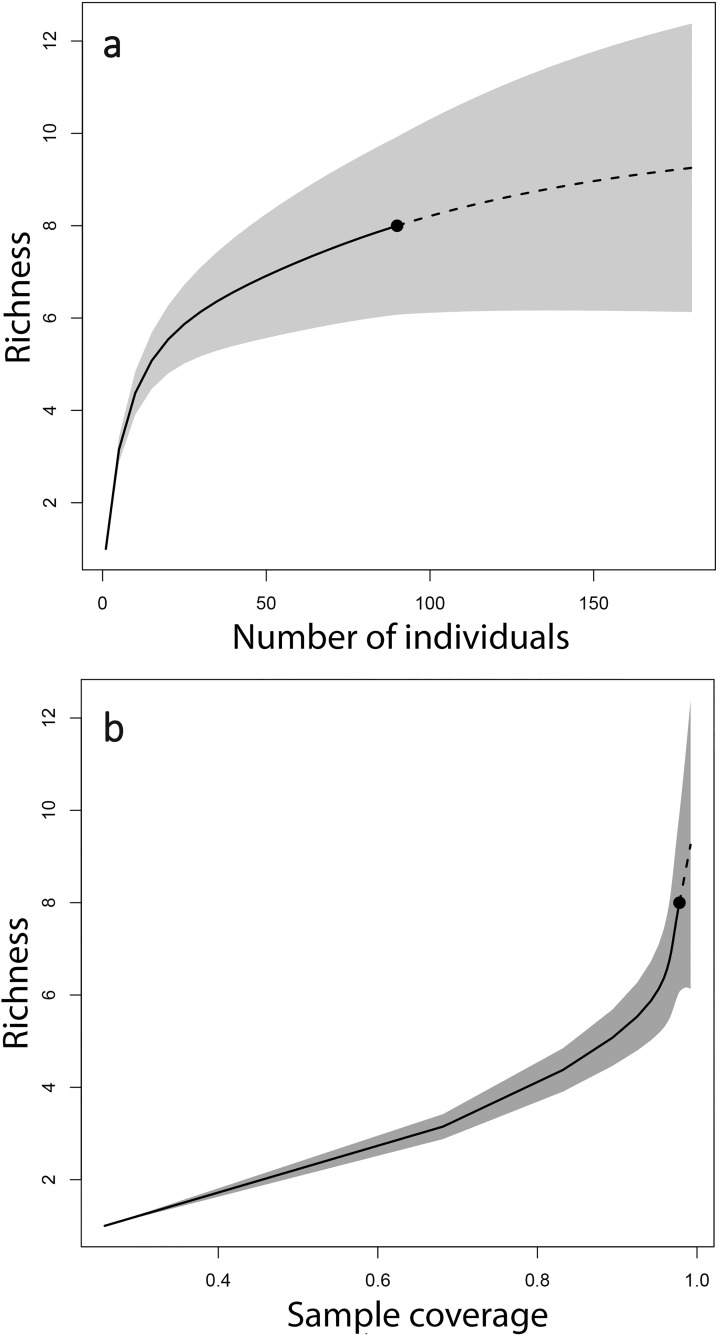
Species richness estimates for a rarefied and extrapolated sample with respect to sample size (a), and estimated species richness according to sample coverage (b). Shaded areas represent 95% confidence intervals, and dashed lines represent extrapolated data. Reprinted from the online version of the software iNEXT (https://chao.shinyapps.io/iNEXT/) under a CC BY license, with permission from Anne Chao, original copyright 2013.

We captured a total of eight species from the families Phyllostomidae (*Sturnira burtonlimi*, *Dermanura tolteca*, *Hylonycteris underwoodi*, and *Anoura cultrata*) and Vespertilionidae (*Lasiurus blossevillii*, *Myotis nigricans*, *M*. *oxyotus* and *M*. *keaysi*). *Sturnira burtonlimi* was the most commonly captured bat, with a relative abundance of 43%, followed by *M*. *oxyotus* (19%) and *M*. *keaysi* (17%). The only two species captured in polyester nets were *S*. *burtonlimi* and *H*. *underwoodi*, whereas all species were captured at least once in monofilament nets. For the five less abundant species we almost exclusively captured male specimens, whilst sex ratio was balanced or female-biased for the three most abundant species (S3 Table). The rarefaction and extrapolation curve for species richness indicates that doubling our sample to 180 would likely increase richness by just one species (Fig 2A). Our sample coverage for the 90 individuals captured and eight species identified with our current effort is 0.97 (Fig 2B), suggesting that the community of understorey bats was adequately sampled.

We sequenced the DNA barcodes (partial COI sequences) of 41 individuals belonging to the eight species. *Myotis* spp. and *Hylonycteris underwoodi* samples were successfully sequenced with both primer sets, whilst Ivanova's Mammal Cocktail [73] provided better results with tissue samples of *Sturnira burtonlimi*, *Dermanura tolteca*, and *Anoura cultrata*. Samples from *A*. *cultrata* and *L*. *blossevilli* showed medium and low quality (S4 Table) and thus, we discarded them from further analyses.

When the obtained sequences were BLASTed, the closest match confirmed the in situ morphological identification of four of the captured species (Table 1). In contrast, the closest BOLD sequence of the specimen we morphologically identified as *Myotis oxyotus* belonged to a *M*. *yumanensis* from California (USA), whereas the only available public sequence assigned to *M*. *oxyotus*, from a bat of Cuzco (Perú), exhibited noticeable genetic difference with our samples (identity 90.0–90.4%; GenBank code JN847707 [81]). In the case of *Sturnira burtonlimi*, the closest sequences found in BOLD were assigned to *Sturnira ludovici*; no sequence for *Sturnira burtonlimi* has hitherto been published in BOLD and GenBank.

**Table 1:** Closest species found in BOLD and GenBank for the sequences of COI gene we got in this study from bats of Valle del Silencio, at Talamanca Range in Costa Rica. When the closest public sequence found is from the same mountain range—marked (*)—, the next closest sequence from a different area has also been shown.

Species sampled	Closest sequence in BOLD	Origin	Identity (%)	GenBank code	Authors
*Myotis keaysi*	*M*. *keaysi*	Chiriquí, Panamá (*)	99–99.9%	JF447424	Clare et al. (2011)
	*M*. *keaysi*	Santa Ana, El Salvador	96–97%	JF446533	Clare et al. (2011)
*M*. *nigricans*	*M*. *nigricans*	Guyana	96.5%	EF080493	Clare et al. (2007)
*M*. *oxyotus*	*M*. *yumanensis*	California, USA	94.4–94.8%	GU723137	Streicker et al. (2010)
*Dermanura tolteca*	*D*. *tolteca*	Ahuachapan, El Salvador	100%	JF446467	Clare et al. (2011)
*Hylonycteris underwoodi*	*H*. *underwoodi*	Chiriquí, Panamá (*)	99.5–99.8%	JF447414	Clare et al. (2011)
	*H*. *underwoodi*	Tortuguero, Costa Rica	90.9–91.1%	JF446599	Clare et al. (2011)
*Sturnira burtonlimi*	*S*. *ludovici*	Chiriquí, Panamá (*)	98.4–100%	JF447436 JF447437 JF447438	Clare et al. (2011)
	*S*. *ludovici*	Santa Ana, El Salvador	94.8–95.1%	JF446555	Clare et al. (2011)

Among the four species in which their closest sequences in BOLD matched the morphological identification, only *M*. *nigricans* showed a difference with their closest sequence greater than 2%, namely the intraspecific sequence divergence expected by the Bradley and Baker criteria [82] (Table 1).

**Fig 3:**
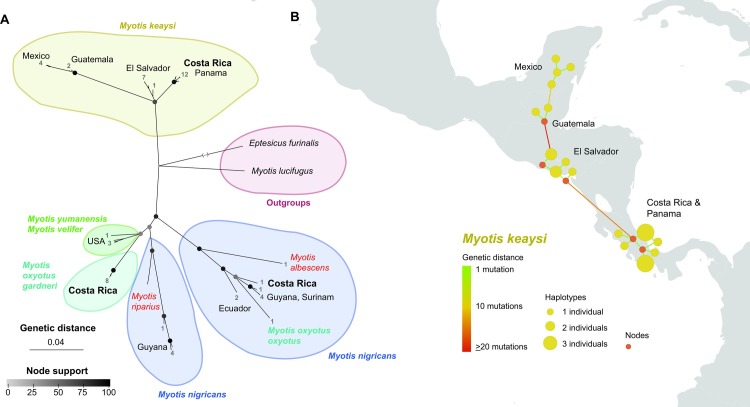
(A) Consensus Bayesian and Maximum Likelihood (ML) radial phylogenetic tree of mitochondrial COI barcode sequences (657 bp) of *Myotis* species. Node colors indicate average values of posterior probabilities (Bayesian) and the bootstrap percentages (ML). Numbers indicate the number of individuals in each tip, which cannot be appreciated due to scale. Note that the branch-length of the output taxon *Eptesicus furinalis* has been cut for scale reasons. A more detailed tree is available as supplementary material. (B) Median-joining network of *Myotis keaysi* haplotypes. Yellow dots indicate actual haplotypes, while red dots missing intermediate haplotypes. Dot size indicates the number of individuals sharing a certain haplotype. The color of connecting lines indicates genetic distance in number of nucleotide polymorphisms. Note that the spatial resolution of the haplotype locations is approximate (country level).

When comparing the sequences obtained with related ones available, the topologies of the built Bayesian and ML trees were consistent both for the *Myotis* sequences and for the phyllostomid ones (S1 and S2 Figs). The *Myotis* tree (Fig 3A and S1 Fig) relates our *M*. *nigricans* specimen to two sequences of the same species, particularly from Guyana and Surinam, but no clear structure is visible among them. Besides, all the sequences we got from *M*. *oxyotus* clustered together, being *M*. *yumanensis* and *M*. *velifer* their closest ones. Notably, the only public sequence attributed to *M*. *oxyotus* in BOLD, mentioned above, appears more closely related to sequences of *M*. *nigricans*—included our own from Valle del Silencio—than to *M*. *oxyotus* from Costa Rica. When we blasted that only public sequence attributed to *M*. *oxyotus* in BOLD, its closest sequences belonged as well to some *M*. *nigricans* from Surinam and Guyana, with identities of about 95%. The sequences of *M*. *keaysi* show a clear geographic structure (Fig 3B): all our samples clustered together along with a single public sequence from the neighbouring Panamá. Sequences from El Salvador form a second group, and those from Guatemala and Mexico a third one. The sequence divergences between those groups are worth mentioning: while the sequences from Costa Rica and Panamá show an average pairwise identity of 99.5±0.26% (1–4 mutational steps estimated in the haplotype network), this value falls to 96.4±0.22% (17–22 mutational steps) when compared with sequences from El Salvador, and down to 91.1±0.53% (66–83 mutational steps) with sequences from Mexico and Guatemala. Pairwise identities between those from El Salvador and Mexico-Guatemala averaged 92.5±0.37% (56–70 mutational steps).

**Fig 4:**
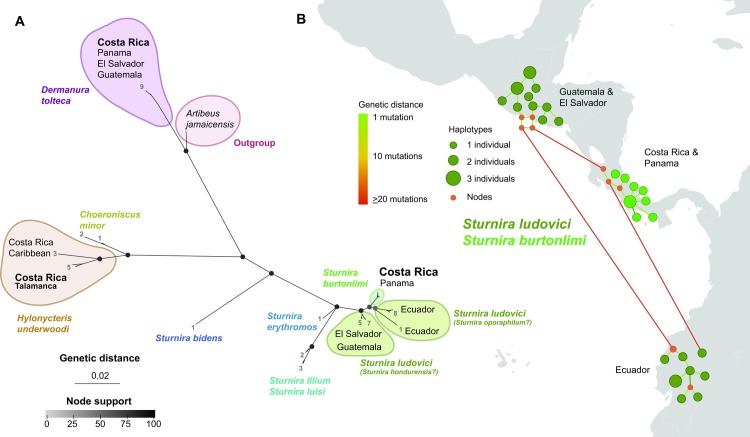
(A) Consensus Bayesian and Maximum Likelihood (ML) radial phylogenetic tree of mitochondrial COI barcode sequences (657 bp) of phyllostomid species. Node colors indicate average values of posterior probabilities (Bayesian) and the bootstrap percentages (ML). Numbers indicate the number of individuals in each tip, which cannot be appreciated due to scale. Note that the branch-length of the output taxon *Artibeus jamaicensis* has been cut for scale reasons. A more detailed tree is available as supplementary material. (B) Median-joining network of *Sturnira burtonlimi* and *Sturnira ludovici sensu lato* haplotypes. Yellow dots indicate actual haplotypes, while red dots missing intermediate haplotypes. Dot size indicates the number of individuals sharing a certain haplotype. The color of connecting lines indicates genetic distance in number of nucleotide polymorphisms. Note that the spatial resolution of the haplotype locations is approximate (country level).

The phyllostomids’ phylogenetic tree (Fig 4A and S2 Fig) shows that our samples of *H*. *underwoodi* cluster with the only one from Panamá available in BOLD, which also comes from the same Talamanca Range (average pairwise identity 98.5±1.45%). Those sequences show significant differences, though, with samples assigned to the same species obtained in the Caribbean coast in Costa Rica (91.2%, SD = 0.2). Besides, the sequences from *S*. *burtonlimi* and those assigned to *S*. *ludovici* in BOLD show marked population structure (Fig 4B). Thus, our sequences of *S*. *burtonlimi* are very similar to those assigned to *S*. *ludovici* from Talamanca Range in Panamá (average pairwise identity 99.4±0.48%). Similarly, the public sequences assigned to *S*. *ludovici* from El Salvador and Guatemala form a second cluster, and those from Ecuador a third one with one single sequence as a sister branch (JN659772). The average pairwise identity of our sequences with those from El Salvador and Guatemala falls to 94.7±0.18% (29–42 mutational steps), and to 94.2±0.39% (29–41 mutational steps) when compared with the samples from Ecuador. Average pairwise identity of sequences from El Salvador-Guatemala and Ecuador is 94.5±0.59% (31–43 mutational steps).

## Discussion

Our data provide the first published description of the bat community inhabiting the mountain cloud forests at the Talamanca Range. The most abundant species we found at Valle del Silencio are considered mountain-adapted species. This is the case of *M*. *oxyotus*, *M*. *keaysi* and *Sturnira burtonlimi*—formerly included within *S*. *ludovici*—, all of them classified as Andean "para-montane" species [83]. Similarly, *D*. *tolteca*, present from Mexico to Panamá, is apparently restricted to mid elevational slopes (previously recorded at 500–2000 masl in Costa Rica [54]); and *A*. *cultrata*, as well, has been reported inhabiting humid mountain cloud forests above 1000 masl, from Costa Rica to Bolivia and Peru [84]. Others, like *M*. *nigricans*, are ubiquitous bats occupying a wide elevational range, from sea level to above 3100 masl [54,85,86]; this seems also the case of *H*. *underwoodi*, whose altitudinal range goes from sea level at Guatemala and Costa Rica, up to above 2000 masl in Mexico and 2600 masl in Costa Rica [44] (but see [87]); and of *L*. *blossevillii*, with a distribution that extends from southern British Columbia through the western U.S., Mexico, Central America, and South America [88], recorded from sea level to above 3100 masl [54].

While our capture methods and small sample area could indicate that our study is not truly representative, we found that the studied community shares many characteristics with other mountain bat guilds in the Neotropics, such as a reduced species richness [22,24–26,34,89,90], low sampling effectiveness [22], nearly equal presence of frugivorous and insectivorous bats [89], and prevalence of the families Phyllostomidae and Vespertilionidae (e.g. [25,90–94]). Reduction of species richness of bats with elevation is a common pattern in mountain areas (e.g. [25,34,89,90,92]). A global analysis aiming to understand the mechanisms ruling the elevational species richness patterns of bats in the tropics showed that species richness was highest where both temperature and water availability were high, and declined as temperature and water availability decreased [95]. Thus, altitudinally decreasing species richness occurs on mountains with wet, warm bases, whilst mountains with dry, arid bases show mid-elevation diversity peaks. In fact, the lower temperatures, more abrupt meteorological changes, lower habitat complexity, and lower productivity at high elevation, make highland environments less profitable—even unsuitable—for most bat species [22,25,30].

Although rarefaction curves suggest an adequate sampling effort, the bat community herein described is still likely incomplete and biased, as a result of the capture method employed. Species foraging in cluttered environments and close to the ground, e.g. fruit-eating and flower-eating phyllostomids [93,96,97], are more prone to be captured by ground-level mist-netting [22,94,96–101]. In contrast, bats flying in open spaces and high altitude from the ground, such as molossids, tend to be underrepresented with mist nets [96,97,102]. We did indeed notice molossid bats flying high above our sampling sites using ultrasound detectors, but we were unable to get reliable identifications from the recordings. It is noteworthy the abundance of insectivorous vespertilionid bats in our captures, as those tend to more easily detect the mist nets in their foraging grounds than frugivorous or nectarivorous ones. This may be partially explained by the higher effectiveness of the type of mistnets we used—made by monofilament and therefore less detectable by bats—but it may also mirror a higher abundance of this group of bats in the study area.

Despite of the inherent limitations of analyzing a short mitochondrial marker [47,103], multiple studies on bats have demonstrated the applicability of DNA barcodes for identifying species (e.g. [44,47], but see [104]). Additionally, although mitochondrial sequences do not provide concluding evidence due to their maternal inheritance and the different spatial behavior of female and male bats [66,105,106], they can be a valuable tool for anticipating the likely existence of cryptic species by detecting deep intraspecific sequence divergences between mitochondrial lineages [41,44,47].

Our preliminary results from Talamanca Range contribute to unveil likely cryptic diversity in bats, providing further support to results from other studies carried out in the Neotropics [41,43–45,60,103,107] and elsewhere [42,46–51]. There are several molecular, morphological and ecological reasons supporting the existence of cryptic species of bats in Talamanca range and other mountains of Central America.

Regarding the status of *Myotis* species, our phylogenetic tree is fully consistent with the previously published Neotropical phylogenies of this genus based on the mitochondrial Cyt-b and the nuclear RAG2 sequences [60]. Among these concordances, it is noteworthy that the only sequence previously analyzed [60] from *M*. *oxyotus* (same specimen from Lima, Peru; voucher FMNH 129208), shared the same position as the only sequence of South American *M*. *oxyotus* in our tree (from Cuzco, Peru; Gen Bank code JN847707), i.e. as a sister species of *M*. *nigricans* and *M*. *albescens*. This concordance advocates discarding misidentification of the South American *M*. *oxyotus* specimens whose sequences were analyzed, and gives consistency to the polyphyly of *M*. *oxyotus*, our tree reveals (Fig 3A). Moreover, such polyphyletic structure fits well the two subespecies described by LaVal [108] based on differences in fur length and color, and relative length of the third metacarpal: *M*. *o*. *oxyotus*, in South America, and *M*. *o*. *gardneri*, in Central America. Our results suggest that *M*. *o*. *gardneri* would in fact be a full species—*Myotis gardneri* LaVal 1973—more closely related to the more septentrional mountain bats *M*. *velifer* and *M*. *yumanensis* than to *M*. *oxyotus* in South America.

Our analyses also depict *M*. *keaysi* as a monophyletic group in Central America, but with geographical structure into three clades (Fig 3B). Based on sequences of Cyt-b, Larsen et al. [60] also describe three lineages of *M*. *keaysi* in Central America, but with some differences. Indeed, our clades from El Salvador and Mexico-Guatemala fit well the lineages "*M*. *cf*. *keaysi* 2" and "*M*. *keaysi*" by Larsen et al. [60]. Contrarily, whilst our third clade with sequences from Talamanca Range is clearly linked to the other two, their "*M*. *cf*. *keaysi* 1" from Honduras was more closely related to *M*. *nigricans* and *M*. *albescens* groups, suggesting that this may correspond to some misidentification. These divergent mitochondrial lineages, and the contrasting elevational patterns and habitats described for *M*. *keaysi sensu lato* in Central America [108,109], are consistent with the occurrence of further cryptic species [24]. Moreover, Mantilla-Meluk and Muñoz-Garay [110] recently proposed the recognition of *M*. *k*. *keaysi* Allen 1914, and *M*. *k*. *pilosatibialis* LaVal 1973 as full species, based on the sympatric populations of both taxa and the presence of unique discrete characters; the former inhabiting mostly South America, and the latter the whole Central America spreading south. Nevertheless, the phylogenetic relation of both groups is still unresolved, mostly due to the lack of molecular information of *M*. *k*. *keaysi*. Finally, the sequences of *M*. *nigricans* depict a paraphyletic species complex as several previous studies already revealed (e.g. [42,59,60]). Further studies will surely describe further cryptic species within this group (e.g. [107]).

Our phyllostomid tree is also consistent with previously published phylogenetic analyses using different mitochondrial and molecular markers [52]. Following Velazco and Patterson [52], the public sequences we analyzed from El Salvador and Guatemala assigned to *S*. *ludovici* would belong to *S*. *hondurensis*, and the two lineages from Ecuador would most likely correspond to *S*. *ludovici* and *S*. *oporaphilum*, whilst those inhabiting in Talamanca Range correspond to *S*. *burtonlimi*. In the case of *S*. *burtonlimi* and *S*. *ludovici sensu lato*, the genetic distances we observed answer to the morphological differences recently described by Velazco and Patterson [61]. Is noteworthy the inconsistency regarding the relation of the single sequence of *S*. *ludovici* from Ecuador, which in the phylogenetic tree appears linked to the other sequences from Ecuador, whilst in the haplotype network is closer to *S*. *burtonlimi* from Talamanca Range. Higher phylogenetic resolution will be necessary to unveil the actual relationships between these sibling species and populations.

Finally, as described by Clare *et al*. [44], *H*. *underwoodi* split into a highland lineage, ecologically coincidental with that described by Thomas [111], and a lowland Caribbean one, which may belong to some new species. These *H*. *underwoodi* clades are the less known ones, and no morphological or ecological differences have hitherto been described, but the limited data on elevational distribution given by Clare et al. [44] suggest that both lineages could have strong ecological separation, which will be worth studying.

Lastly, the noticeable genetic distances of the samples of *M*. *oxytous*, *M*. *keaysi*, *S*. *burtonlimi* and *H*. *underwoodi* from the Talamanca range—focusing only on the species with a larger sample size and better sequencing quality—with their conspecifics in other mountain ranges of Central and South America depicts geographic isolation, which underlines the importance for speciation and endemicity of these mountains of Costa Rica and Panamá. This is congruent, firstly, with the character of hotspots of species richness and endemism of mountain regions for many taxa, especially in the tropics [14,15,36], and secondly with the geographic location of the Talamanca Range. Our results further suggest that even highly mobile taxa, such as bats, may equally suffer from isolation in upper elevations as other less vagile organisms, likely promoting speciation in the long term and contributing to the high levels of endemicity within these ecosystems.

In the current scenario of strong global warming [112], the survival of species restricted to mountain environments is severely threatened. The upward shift of vegetation communities already documented and going on in tropical areas [11,113] will severely affect the animals inhabiting mountain regions [114,115]. The effect will be stronger for species less tolerant to temperature changes, as bats seem to be [22,34,116]. Moreover, the extinction risk will be particularly high in medium sized isolated mountain systems like the Talamanca range, where land availability dramatically decreases with elevation and not much range is left upward. In this context, further research to set the taxonomic status of the Neotropical mountain bats is urgent. Preliminary phylogenetic screenings such as this study should give rise to more detailed analyses with multiple mitochondrial and nuclear markers and accompanied by differences in other traits regarding morphology, ecology, echolocation, etc., to describe genetically distinct populations as unambiguously belonging to different species [47,117]. That knowledge would help us identify the key environmental factors for the survival of mountain bats, and monitor the evolution of their populations in the following decades.

## Supporting Information

S1 Fig Consensus Bayesian tree generated in MrBayes 3.2.5 (Huelsenbeck & Ronquist, 2001; Ronquist et al. 2012) from sequences of mitochondrial COI gene (657 bp) of *Myotis* species, applying the substitution model HKY+I. The numbers of each node respectively indicate the posterior probability and the bootstrap percentage in the analogous ML tree, separated by a semicolon (when both values are equal one single value is given). Scale bar units are substitution per site. Sequences from Valle del Silencio are marked "VS" after the code number; public sequences are marked "gb" followed by their Genbank accession number. The first characters of each code identify the species to which authors assigned the sample: Efu means *Eptesicus furinalis* (outgroup); Mal, *Myotis albescens*; Mke *keaysi*; Mlu, *M*. *lucifugus*; Mni, *M*. *nigricans*; Mox, *M*. *oxyotus*; Mri, *M*. *riparius*; Mve, *M*. *velifer*; Myu, *M*. *yumanensis*; The black arrows points out the only sequence of *M*. *nigricans* from Valle del Silencio, and the only available public sequence of COI assigned to *M*. *oxyotus*, from Peru, which clusters with sequences of *M*. *nigricans*, instead of with those of *M*. *oxyotus* from Costa Rica and Panama.
(TIF)

S2 Fig Consensus Bayesian tree generated in MrBayes 3.2.5 (Huelsenbeck & Ronquist, 2001; Ronquist et al. 2012) from sequences of mitochondrial COI gene (657 bp) of phyllostomid species, applying the substitution model HKY+I. The numbers of each node respectively indicate the posterior probability and the bootstrap percentage in the analogous ML tree, separated by a semicolon (when both values are equal one single value is given). Scale bar units are substitution per site. Sequences from Valle del Silencio are marked "VS" after the code number; public sequences are marked "gb" followed by their Gen Bank accession number. The first characters of each code identify the species to which authors assigned the sample: Aja means *Artibeus jamaicensis* (outgroup); Dto, *Dermanura tolteca*; Csp, *Choeroniscus sp*.; Cmi, *C*. *minor*; Hun, *Hylonycteris underwoodi*; Sbi, *Sturnira bidens;* Sbu, *S*. *burtonlimi*; Ser, *S*. *erythromos*; Sli, *S*. *lilium*; Slu, *S*. *ludovici*; Sls, *S*. *luisi*.
(TIF)

S1 Table Sequences obtained from each species with the two sets of primers used, namely, UTyr and C1L705 (Hassanin et al. 2012), and the cocktail C_VF1LFt1+C_VR1LRt1 (Ivanova et al. 2012). For each set of primers we respectively indicate de amount of samples analysed, in how many of them we got the whole fragment of COI sequenced (657 bps), and the sequences obtained with high quality (HQ) reads at more than 90% of the bps.
(DOCX)

S2 Table List of public sequences of the mitochondrial DNA gen cytochrome oxidase I (COI) used in this study for comparison. (DOCX)

S3 Table Number of males and females captured for each species. (DOCX)

S4 Table List of sequences of the mitochondrial DNA gene cytochrome oxidase I (COI) obtained from bats at Valle del Silencio (Costa Rica) and uploaded to GenBank. (DOCX)
