# Supplementary figures and images for: Unveiling the Hidden Bat Diversity of a Neotropical Montane Forest
Source: PLoS One. 2016 Oct 5;11(10):e0162712. doi: 10.1371/journal.pone.0162712 (PMC5051729; doi:10.1371/journal.pone.0162712)

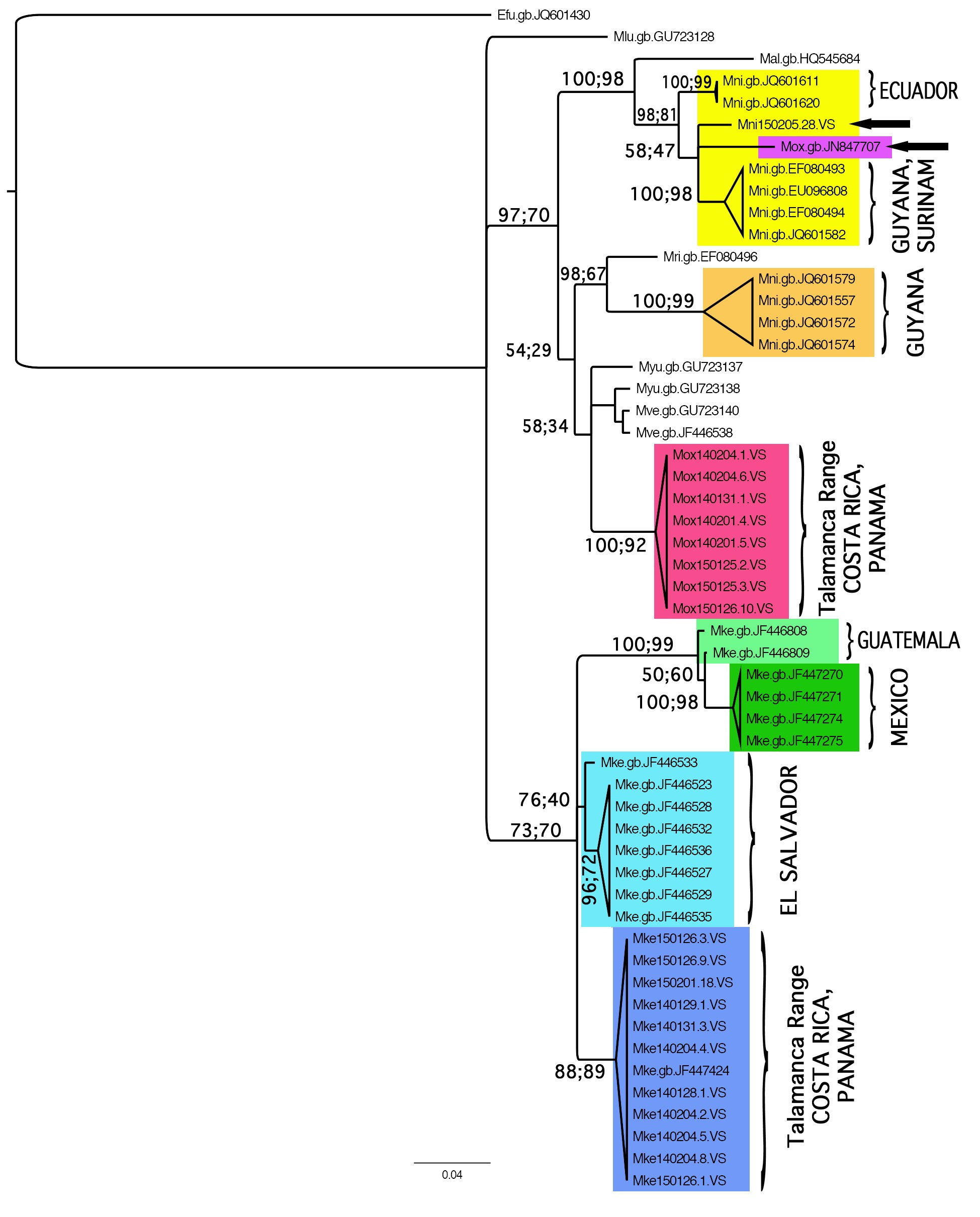

Supplement: S1 Fig — The numbers of each node respectively indicate the posterior probability and the bootstrap percentage in the analogous ML tree, separated by a semicolon (when both values are equal one single value is given). Scale bar units are substitution per site. Sequences from Valle del Silencio are marked "VS" after the code number; public sequences are marked "gb" followed by their Genbank accession number. The first characters of each code identify the species to which authors assigned the sample: Efu means Eptesicus furinalis (outgroup); Mal, Myotis albescens; Mke keaysi; Mlu, M. lucifugus; Mni, M. nigricans; Mox, M. oxyotus; Mri, M. riparius; Mve, M. velifer; Myu, M. yumanensis; The black arrows points out the only sequence of M. nigricans from Valle del Silencio, and the only available public sequence of COI assigned to M. oxyotus, from Peru, which clusters with sequences of M. nigricans, instead of with those of M. oxyotus from Costa Rica and Panama. (TIF) [file pone.0162712.s001.tif]

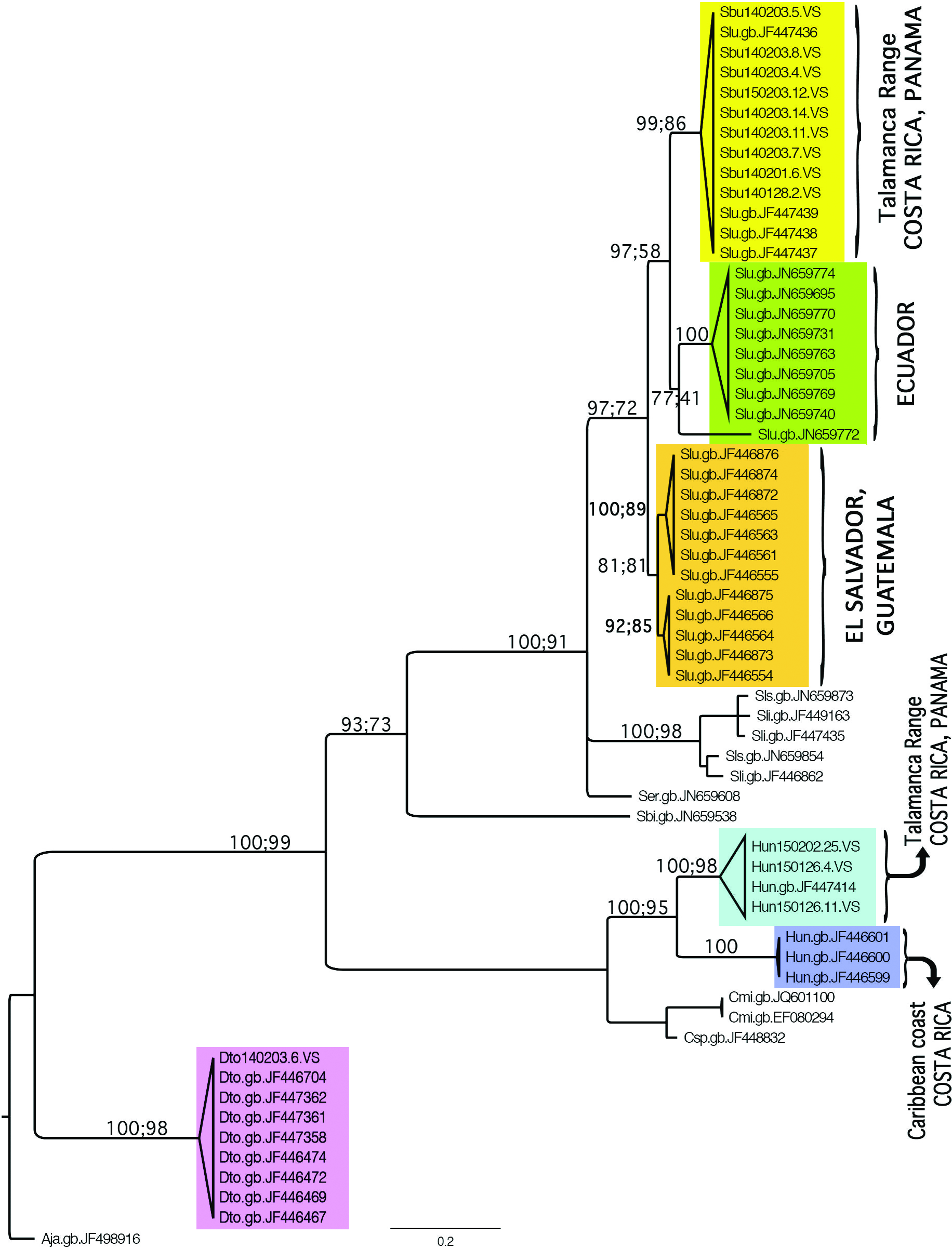

Supplement: S2 Fig — The numbers of each node respectively indicate the posterior probability and the bootstrap percentage in the analogous ML tree, separated by a semicolon (when both values are equal one single value is given). Scale bar units are substitution per site. Sequences from Valle del Silencio are marked "VS" after the code number; public sequences are marked "gb" followed by their Gen Bank accession number. The first characters of each code identify the species to which authors assigned the sample: Aja means Artibeus jamaicensis (outgroup); Dto, Dermanura tolteca; Csp, Choeroniscus sp.; Cmi, C. minor; Hun, Hylonycteris underwoodi; Sbi, Sturnira bidens; Sbu, S. burtonlimi; Ser, S. erythromos; Sli, S. lilium; Slu, S. ludovici; Sls, S. luisi. (TIF) [file pone.0162712.s002.tif]
