# Supplementary material for: Unveiling the Hidden Bat Diversity of a Neotropical Montane Forest
Source: PLoS One. 2016 Oct 5;11(10):e0162712. doi: 10.1371/journal.pone.0162712 (PMC5051729; doi:10.1371/journal.pone.0162712)
Supplement: S1 Table — For each set of primers we respectively indicate de amount of samples analysed, in how many of them we got the whole fragment of COI sequenced (657 bps), and the sequences obtained with high quality (HQ) reads at more than 90% of the bps. (DOCX) [file pone.0162712.s003.docx]

**S1 Table:** Sequences obtained from each species with the two sets of primers used, namely, UTyr and C1L705 (Hassanin et al. 2012), and the cocktail C_VF1LFt1+C_VR1LRt1 (Ivanova et al. 2012). For each set of primers we respectively indicate de amount of samples analysed, in how many of them we got the whole fragment of COI sequenced (657 bps), and the sequences obtained with high quality (HQ) reads at more than 90% of the bps.

| Species | Hassanin et al. 2012 | | | Ivanova et al. 2012 | | |
| --- | --- | --- | --- | --- | --- | --- |
|  | Samples analyzed | Sequences obtained | HQ  sequences | Samples analyzed | Sequences obtained | HQ sequences |
| *Lasiurus blossevillii* | 6 | 1 | 0 | 6 | 1 | 0 |
| *Myotis keaysi* | 11 | 11 | 10 | 11 | 11 | 10 |
| *M. nigricans* | 1 | 1 | 1 | 1 | 1 | 1 |
| *M. oxyotus* | 10 | 9 | 8 | 10 | 9 | 8 |
| *Anoura cultrata* | 1 | 1 | 0 | 1 | 0 | 0 |
| *Dermanura tolteca* | 1 | 1 | 1 | 1 | 0 | 0 |
| *Hylonycteris underwoodi* | 7 | 5 | 5 | 7 | 6 | 6 |
| *Sturnira burtonlimi* | 12 | 5 | 1 | 12 | 10 | 9 |
