# Supplementary material for: Unveiling the Hidden Bat Diversity of a Neotropical Montane Forest
Source: PLoS One. 2016 Oct 5;11(10):e0162712. doi: 10.1371/journal.pone.0162712 (PMC5051729; doi:10.1371/journal.pone.0162712)
Supplement: S2 Table — (DOCX) [file pone.0162712.s004.docx]

**S2 Table:** List of public sequences of the mitochondrial DNA gen cytochrome oxidase I (COI) used in this study for comparison.

| **GenBank code** | **Species** | **source** | **Authors** |
| --- | --- | --- | --- |
| JF498916 | *Artibeus jamaicensis* | Belize | Clare, E. L. (unpubl.) |
| JF446704 | *Dermanura tolteca* | Guatemala | Clare et al. (2011) |
| JF447362 | *D. tolteca* | Chiriquí, Panama | Clare et al. (2011) |
| JF447361 | *D. tolteca* | Chiriquí, Panama | Clare et al. (2011) |
| JF447358 | *D. tolteca* | Altos de Campana, Panama | Clare et al. (2011) |
| JF446474 | *D. tolteca* | El Salvador | Clare et al. (2011) |
| JF446472 | *D. tolteca* | El Salvador | Clare et al. (2011) |
| JF446469 | *D. tolteca* | El Salvador | Clare et al. (2011) |
| JF446467 | *D. tolteca* | El Salvador | Clare et al. (2011) |
| EF080294 | *Choeroniscus minor* | Guyana | Clare et al. (2007) |
| JQ601100 | *C. minor* | Surinam | Engstrom et al. (unpubl.) |
| JF448832 | *C. sp.* | Napo, Ecuador | Clare et al. (2011) |
| JQ601430 | *Eptesicus furinalis* | Surinam | Lim & Arcilla (unpubl.) |
| JF446599 | *Hylonycteris underwoodi* | Caribbean coast, Costa Rica | Clare et al. (2011) |
| JF446600 | *H. underwoodi* | Caribbean coast, Costa Rica | Clare et al. (2011) |
| JF446601 | *H. underwoodi* | Caribbean coast, Costa Rica | Clare et al. (2011) |
| JF447414 | *H. underwoodi* | Chiriquí, Panama | Clare et al. (2011) |
| HQ545684 | *Myotis albescens* | Surinam | Lim et al. (unpubl.) |
| JF446523 | *Myotis keaysi* | El Salvador | Clare et al. (2011) |
| JF446527 | *M. keaysi* | El Salvador | Clare et al. (2011) |
| JF446528 | *M. keaysi* | El Salvador | Clare et al. (2011) |
| JF446529 | *M. keaysi* | El Salvador | Clare et al. (2011) |
| JF446532 | *M. keaysi* | El Salvador | Clare et al. (2011) |
| JF446533 | *M. keaysi* | El Salvador | Clare et al. (2011) |
| JF446535 | *M. keaysi* | El Salvador | Clare et al. (2011) |
| JF446536 | *M. keaysi* | El Salvador | Clare et al. (2011) |
| JF446808 | *M. keaysi* | Guatemala | Clare et al. (2011) |
| JF446809 | *M. keaysi* | Guatemala | Clare et al. (2011) |
| JF447270 | *M. keaysi* | Mexico | Clare et al. (2011) |
| JF447271 | *M. keaysi* | Mexico | Clare et al. (2011) |
| JF447274 | *M. keaysi* | Mexico | Clare et al. (2011) |
| JF447275 | *M. keaysi* | Mexico | Clare et al. (2011) |
| JF447424 | *M. keaysi* | Chiriquí, Panama | Clare et al. (2011) |
| GU723128 | *Myotis lucifugus* | Indiana, USA | Streicker et al. (2010) |
| JQ601557 | *Myotis nigricans* | Guyana | Engstrom et al. (unpubl.) |
| JQ601572 | *M. nigricans* | Guyana | Lim et al. (unpubl.) |
| JQ601574 | *M. nigricans* | Guyana | Lim et al. (unpubl.) |
| JQ601579 | *M. nigricans* | Guyana | Lim et al. (unpubl.) |
| JQ601611 | *M. nigricans* | Yasuní, Ecuador | Engstrom et al. (unpubl.) |
| JQ601620 | *M. nigricans* | Orellana, Ecuador | Reid et al. (unpubl.) |
| JQ601582 | *M. nigricans* | Guyana | Lim et al. (unpubl.) |
| EF080493 | *M. nigricans* | Ecuador | Clare et al. (2007) |
| EF080494 | *M. nigricans* | Guyana | Clare et al. (2007) |
| EU096808 | *M. nigricans* | Surinam | Borisenko et al. (2008) |
| JN847707 | *Myotis oxyotus* | Cuzco, Peru | Taylor et al. 2011 |
| EF080496 | *Myotis riparius* | Guyana | Clare et al. (2007) |
| GU723140 | *Myotis velifer* | Arizona, USA | Streicker et al. (2010) |
| JF446538 | *M. velifer* | El Salvador | Clare et al. (2011) |
| GU723138 | *Myotis yumanensis* | Arizona, USA | Streicker et al. (2010) |
| GU723137 | *M. yumanensis* | California, USA | Streicker et al. (2010) |
| JN659538 | *Sturnira bidens* | Pichincha, Ecuador | Jarrin & Clare (unpubl.) |
| JN659608 | *Sturnira erythromos* | Pichincha, Ecuador | Jarrin & Clare (unpubl.) |
| JF449163 | *Sturnira lilium* | Napo, Ecuador | Clare et al. (2011) |
| JF447435 | *S. lilium* | Darien, Panama | Clare et al. (2011) |
| JF446862 | *S. lilium* | Guatemala | Clare et al. (2011) |
| JF447437 | *Sturnira ludovici* | Chiriquí, Panama | Clare et al. (2011) |
| JF447438 | *S. ludovici* | Chiriquí, Panama | Clare et al. (2011) |
| JF447439 | *S. ludovici* | Chiriquí, Panama | Clare et al. (2011) |
| JF447436 | *S. ludovici* | Chiriquí, Panama | Clare et al. (2011) |
| JF446554 | *S. ludovici* | El Salvador | Clare et al. (2011) |
| JF446873 | *S. ludovici* | Guatemala | Clare et al. (2011) |
| JF446564 | *S. ludovici* | El Salvador | Clare et al. (2011) |
| JF446566 | *S. ludovici* | El Salvador | Clare et al. (2011) |
| JF446875 | *S. ludovici* | Guatemala | Clare et al. (2011) |
| JF446555 | *S. ludovici* | El Salvador | Clare et al. (2011) |
| JF446561 | *S. ludovici* | El Salvador | Clare et al. (2011) |
| JF446563 | *S. ludovici* | El Salvador | Clare et al. (2011) |
| JF446565 | *S. ludovici* | El Salvador | Clare et al. (2011) |
| JF446872 | *S. ludovici* | Guatemala | Clare et al. (2011) |
| JF446874 | *S. ludovici* | Guatemala | Clare et al. (2011) |
| JF446876 | *S. ludovici* | Guatemala | Clare et al. (2011) |
| JN659740 | *S. ludovici* | Pastaza, Ecuador | Jarrin & Clare (unpubl.) |
| JN659769 | *S. ludovici* | Tungurahua, Ecuador | Jarrin & Clare (unpubl.) |
| JN659705 | *S. ludovici* | Napo, Ecuador | Jarrin & Clare (unpubl.) |
| JN659763 | *S. ludovici* | Sucumbios, Ecuador | Jarrin & Clare (unpubl.) |
| JN659695 | *S. ludovici* | Tungurahua, Ecuador | Jarrin & Clare (unpubl.) |
| JN659774 | *S. ludovici* | Zamora Chinchipe, Ecuador | Jarrin & Clare (unpubl.) |
| JN659731 | *S. ludovici* | Tungurahua, Ecuador | Jarrin & Clare (unpubl.) |
| JN659770 | *S. ludovici* | Napo, Ecuador | Jarrin & Clare (unpubl.) |
| JN659772 | *S. ludovici* | Pichincha, Ecuador | Jarrin & Clare (unpubl.) |
| JN659873 | *Sturnira luisi* | Esmeraldas, Ecuador | Jarrin & Clare (unpubl.) |
| JN659854 | *S. luisi* | Pichincha, Ecuador | Jarrin & Clare (unpubl.) |
