# Supplementary material for: Unveiling the Hidden Bat Diversity of a Neotropical Montane Forest
Source: PLoS One. 2016 Oct 5;11(10):e0162712. doi: 10.1371/journal.pone.0162712 (PMC5051729; doi:10.1371/journal.pone.0162712)
Supplement: S3 Table — (DOCX) [file pone.0162712.s005.docx]

**S3 Table:** Number of males and females captured for each species.

| Species | Males | Females |
| --- | --- | --- |
| *Lasiurus blossevillii* | 7 | 0 |
| *Myotis keaysi* | 9 | 6 |
| *Myotis nigricans* | 2 | 0 |
| *Myotis oxyotus* | 3 | 14 |
| *Anoura cultrata* | 1 | 0 |
| *Dermanura tolteca* | 0 | 1 |
| *Hylonycteris underwoodi* | 7 | 1 |
| *Sturnira burtonlimi* | 16 | 23 |
