# Supplementary material for: Unveiling the Hidden Bat Diversity of a Neotropical Montane Forest
Source: PLoS One. 2016 Oct 5;11(10):e0162712. doi: 10.1371/journal.pone.0162712 (PMC5051729; doi:10.1371/journal.pone.0162712)
Supplement: S4 Table — (DOCX) [file pone.0162712.s006.docx]

**S4 Table:** List of sequences of the mitochondrial DNA gene cytochrome oxidase I (COI) obtained from bats at Valle del Silencio (Costa Rica) and uploaded to GenBank.

| **Species** | **Sequence code** | **GenBank code** | **bp length** | **HQ%** |
| --- | --- | --- | --- | --- |
| *Myotis oxyotus* | Mox140201.4.VS | KX814406 | 657 | 91.60% |
|  | Mox140204.6.VS | KX814409 | 657 | 99.25% |
|  | Mox140201.5.VS | KX814407 | 657 | 96% |
|  | Mox140131.1.VS | KX814405 | 657 | 99.70% |
|  | Mox140204.1.VS | KX814408 | 657 | 97.90% |
|  | Mox150125.2.VS | KX814410 | 657 | 99.20% |
|  | Mox150125.3.VS | KX814411 | 657 | 91.60% |
|  | Mox150126.10.VS | KX814412 | 657 | 99.10% |
| *Myotis keaysi* | Mke140204.2.VS | KX814396 | 657 | 93.90% |
|  | Mke140204.4.VS | KX814397 | 657 | 97.10% |
|  | Mke140204.5.VS | KX814398 | 657 | 98.50% |
|  | Mke140204.8.VS | KX814399 | 657 | 88.00% |
|  | Mke150126.1.VS | KX814400 | 657 | 97.90% |
|  | Mke150201.18.VS | KX814403 | 657 | 92.50% |
|  | Mke140128.1.VS | KX814393 | 657 | 98.90% |
|  | Mke150126.3.VS | KX814401 | 657 | 93.80% |
|  | Mke140129.1.VS | KX814394 | 657 | 94.50% |
|  | Mke140131.3.VS | KX814395 | 657 | 91.90% |
|  | Mke150126.9.VS | KX814402 | 657 | 93.80% |
| *Myotis nigricans* | Mni150205.28.VS | KX814404 | 657 | 99.80% |
| *Hylonycteris underwoodi* | Hun150126.11.VS | KX814391 | 657 | 93.50% |
|  | Hun150126.4.VS | KX814390 | 657 | 97.70% |
|  | Hun150202.25.VS | KX814392 | 657 | 96.20% |
| *Dermanura tolteca* | Dto140203.6.VS | KX814389 | 657 | 90.40% |
| *Sturnira burtonlimi* | Sbu140201.6.VS | KX814414 | 657 | 93.90% |
|  | Sbu140203.11.VS | KX814419 | 657 | 94.40% |
|  | Sbu140203.4.VS | KX814415 | 657 | 95.30% |
|  | Sbu140203.5.VS | KX814416 | 657 | 95% |
|  | Sbu140203.7.VS | KX814417 | 657 | 93.40% |
|  | Sbu140128.2.VS | KX814413 | 657 | 90.30% |
|  | Sbu140203.8.VS | KX814418 | 657 | 98.60% |
|  | Sbu140203.14.VS | KX814420 | 657 | 92.40% |
|  | Sbu150203.12.VS | KX814421 | 657 | 93.20% |
